# Novel genetic variants in tuberculosis-associated immune reconstitution inflammatory syndrome

**DOI:** 10.1093/ajrccm/aamag037

**Published:** 2026-06-01

**Authors:** Cecilia Wieder, Stuart Horswell, Gavin Kelly, Cari Stek, Raymond Moseki, Peter Rossi-Smith, Timothy MD Ebbels, Robert J Wilkinson, Graeme Meintjes, Rachel PJ Lai

**Affiliations:** 1 Section of Bioinformatics, Division of Systems Medicine, Department of Metabolism, Digestion and Reproduction, Imperial College London, London, UK; 2 Bioinformatics and Biostatistics Science Technology Platform, The Francis Crick Institute, London, UK; 3 Wellcome Discovery Research Platforms in Infection, Centre for Infectious Disease Research in Africa, Infectious Disease and Molecular Medicine and Department of Medicine, University of Cape Town, Observatory, South Africa; 4 Section of Adult Infectious Disease, Department of Infectious Disease, Imperial College London, London, UK; 5 The Francis Crick Institute, London, UK; 6 Department of Medicine, University of Cape Town, Observatory, South Africa; 7 Blizard Institute, Queen Mary University of London, London, UK

## To the Editor

Patients with HIV-associated tuberculosis (TB) are at risk of developing immune reconstitution inflammatory syndrome (IRIS), a self-limiting yet severe hyperinflammatory condition occurring shortly after the initiation of antiretroviral therapy (ART) (1, 2). The pathogenesis is mediated by dysregulated innate and adaptive immune responses. Specifically, the rapid restoration of CD4+ T cell function following ART might amplify myeloid cell activation, triggering exaggerated inflammatory responses against mycobacterial antigens (3, 4). While the clinical manifestations and immunopathology are well-characterised, the genetic determinants underlying TB-IRIS susceptibility remain poorly defined.

Initial genetic insights into TB-IRIS came from candidate gene studies identifying associations with variants in inflammatory cytokines like *IL6* and *TNFA* and the immune-related gene *SLC11A1* (5, 6). Complementary work highlighted associations with specific combinations of *KIR* and *HLA* alleles (7). More recently, targeted sequencing suggested a role for rare, protein-altering variants in genes linked to primary hemophagocytic lymphohistiocytosis in mycobacterial-IRIS (8). However, conflicting results regarding *LTA4H* promoter polymorphisms underscore potential ethnic variation in genetic susceptibility and the need for further investigation across diverse populations (9, 10).

Given the substantial burden of HIV-1 and TB and the elevated incidence of TB-IRIS in sub-Saharan Africa, a better understanding of genetic determinants is important for understanding pathogenesis and potential predisposition to this inflammatory condition. In this study, we performed whole-exome sequencing (WES) on South African patients from the PredART trial (11), consisting of 82 individuals with advanced HIV-associated TB (46 TB-IRIS cases vs. 36 non-IRIS controls; Table 1). Compared with non-IRIS patients, those with TB-IRIS had significantly lower baseline CD4 counts (median = 31 vs. 65 cells/µL; p=0.015). To enrich our analysis for functionally relevant loci, we integrated complementary RNA-sequencing data that identified 5,918 differentially expressed genes (DEGs). We filtered the complete variant dataset (2,871,739 variants) to retain only those located within or immediately flanking these DEGs, resulting in a high-confidence set of 686,067 variants for downstream analysis. The mean number of variants per participant did not differ between TB-IRIS and non-IRIS groups (82,698 vs. 81,296; p=0.7), nor did the overall proportion of SnpEff-defined functional variant classes.

**Figure 1:**
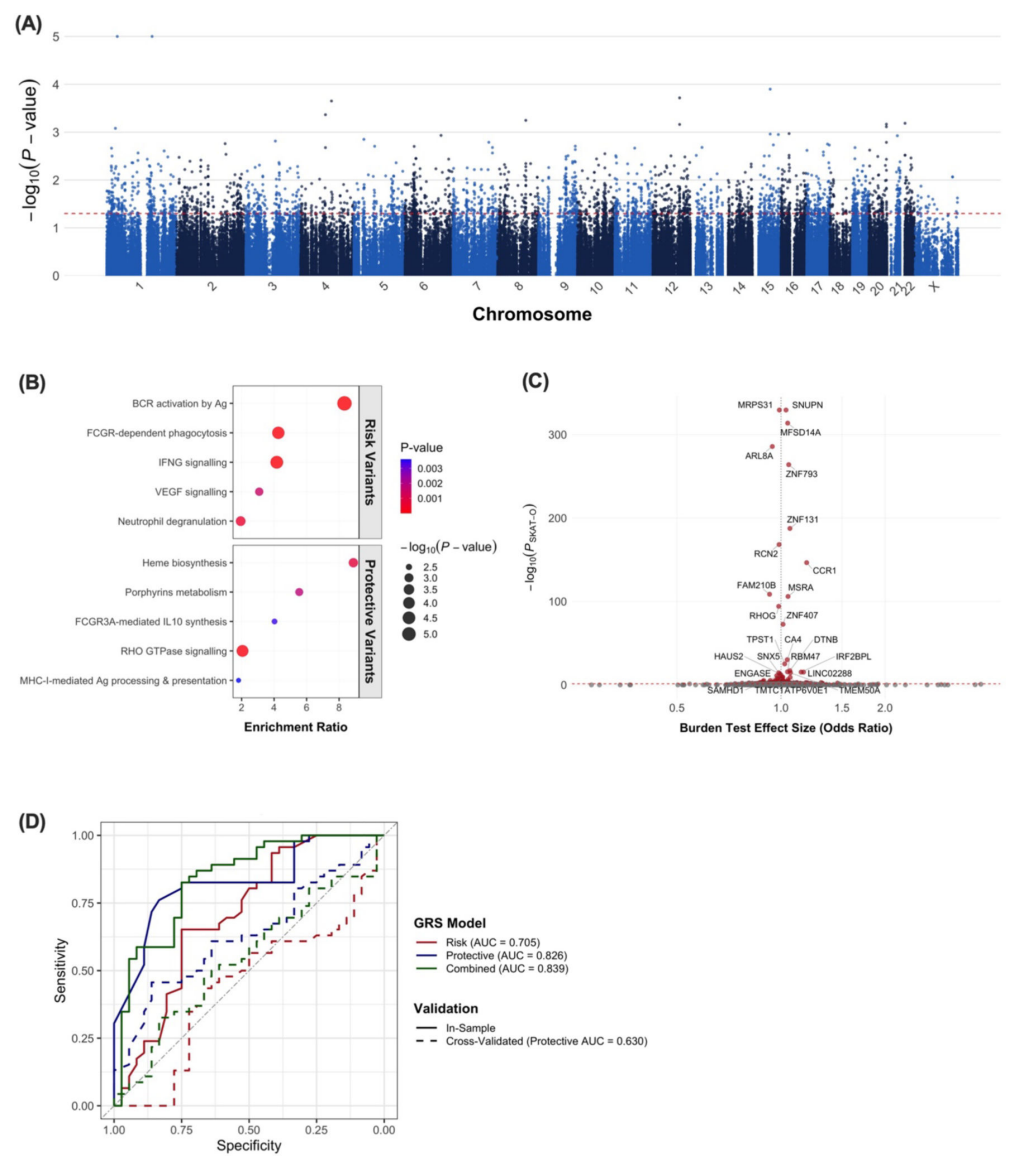
Genetic analysis of TB-IRIS susceptibility using whole exome sequencing. Genomic DNA were extracted from peripheral blood cells from 82 patients with HIV-associated TB, 46 of whom developed TB-IRIS. Samples were sequenced to an average depth of 100x coverage per base. WES data was processed using nf-core/sarek pipeline. Variant calling for single nucleotide variants (SNVs) and small insertions/deletions (indels) was performed and annotated using snpEff to predict functional impacts. Differentially expressed genes (n=5,918) from complementary RNA-sequencing were used to enrich for variants with probable functional impact. **(A)** Manhattan plot of the single-variant association analysis of 686,067 variants within DEG-related regions. The y-axis shows the -log10(p-value) from logistic regression for each variant's association with TB-IRIS status. The horizontal dashed line represents the threshold for nominal significance (p=0.05). **(B)** Pathway over-representation analysis for nominally significant risk variants (top) and protective variants (bottom). The size of the bubble corresponds with the significance of the enrichment. **(C)** Gene-level association plot showing the genetic burden effect size from the Firth’s Burden test (odds ratios, x-axis) and the significance from the SKAT-O test (log_10_(p-value), y-axis). The top 25 most significant genes are labelled. **(D)** Predictive performance of the 5-variant Genetic Risk Score (GRS). The area under the curve (AUC) value indicates the ability of each GRS model to discriminate TB-IRIS cases from non-IRIS controls. Solid lines represent in-sample analysis and dashed lines represent 10-fold cross-validation.

To identify individual variants associated with TB-IRIS, a penalised logistic regression was applied across all 686,067 variants, adjusting for sex and CD4 count as covariates. A total of 1,980 variants mapping to 1,036 unique DEGs were nominally associated with TB-IRIS (p≤0.05) (Figure 1A), but no variants reached genome-wide significance (p≤5x10^-8^). Among the top risk-associated alternate variants were single nucleotide variants (SNVs) in the inflammatory gene *SLCO3A1* (rs2239295 (T>C); OR=7.77), pro-apoptosis mediator *BID* (rs181404 (G>C); OR=7.71), and antiviral gene *HERC5* (rs7699006 (A>G); OR=6.11). Conversely, SNVs in pro-apoptosis gene *FADD* (rs1131677 (A>G); OR=0.19), *HLA-DRB1* (rs28732255 (T>A); OR=0.17), and cytokine gene *IL27* (rs40831 (A>G); OR=0.15) showed strong protective associations. Over-representation analysis of all 1,980 nominally significant variants revealed that TB-IRIS risk variants were significantly enriched in pathways including B cell receptor activation, FcγR-dependent phagocytosis, IFN-γ signalling, and neutrophil degranulation, while protective variants were associated with heme biosynthesis, IL-10 synthesis, and class I MHC pathways (Figure 1B). The enrichment of risk variants in pathways related to humoral immunity highlights a previously underappreciated role for antibody-mediated responses in driving TB-IRIS pathogenesis.

Since complex inflammatory conditions are often polygenic, we next performed a gene-level burden analysis to assess the collective impact of all variants within each DEG. For each of the 5,918 genes, the optimal unified Sequence Kernel Association Test (SKAT-O) (12) was performed, adjusting for sex and CD4 count. The SKAT-O analysis, which combines burden and variance-component tests, identified 428 genes nominally associated with TB-IRIS (p≤0.05) (Figure 1C). Pathway analysis of these genes revealed a significant accumulation of genetic burden in key metabolic pathways, including glucose metabolism (*ADPGK, GAPDH, NUP160, PC, PFKFB2, PGAM1, SLC25A1*) and lipoxin biosynthesis (*ALOX12, ALOX5AP*). Lipoxins are pro-resolving eicosanoids that negatively regulate TB-protective Th1 responses (13), suggesting altered lipid mediator biosynthesis could be a contributing factor in TB-IRIS. Additionally, genes related to mitochondrial translation (*AURKAIP1, MRPL35, MRPS7, MRPS17, MRPS31, MRPS35*) and inflammasome regulation (*ABCA1, AHR, CASP4, MAPK3, NR1D1, SEPTIN4, VIM*) were also significantly burdened. This genetic finding corroborates with previous transcriptomic findings by our group and others on the central role of inflammasome-driven pathogenesis, implicating a previously underappreciated role of immunometabolism in TB-IRIS pathogenesis.

Finally, we assessed the overall genetic burden at the participant level. The total burden score, summed across all 5,918 DEGs, did not differ between groups (p=0.59), suggesting that TB-IRIS risk is not caused by a general increase in the number of genetic variants, but rather by the specific effects of a key set of variants acting together. To explore the potential predictive ability of our findings, we computed two 5-variant Genetic Risk Scores (GRS). The GRS composed of top risk variants showed moderate ability to discriminate cases from controls (AUC = 0.705), while the protective GRS (AUC = 0.826) and combined GRS (AUC = 0.839) performed better (Figure 1D). In the absence of an independent test set, we estimated GRS performance by a 10-fold cross-validation, where genetic risk scores were calculated for each participant using variant weights derived independently from the other 90% of the cohort. The protective GRS retained a modest but significant predictive ability (AUC = 0.630, 95% CI: 0.510–0.751) in discriminating TB-IRIS from non-IRIS (Figure 1D).

Our exome-wide analysis provides novel insights into the complex genetic architecture of TB-IRIS. We acknowledge our sample size is modest, which restricts statistical power to detect variants with small effects. To address this, we integrated functional genomics data by focusing on variants within DEGs, thereby enriching for loci with a higher prior probability of being functionally relevant. Although no single variant achieved genome-wide significance, we identified numerous variants and genes with nominal significance whose cumulative burden across diverse immune and metabolic pathways may collectively contribute to the phenotype. Although our exploratory GRS models have limited predictive performance, they nonetheless support a polygenic contribution to TB-IRIS susceptibility. An unexpected finding of our study was the enrichment of risk variants related to humoral immunity. The roles of B cells and antibodies in TB are complex, with evidence for both protective and detrimental functions (14). The rapid T cell reconstitution following ART might enhance T cell – B cell interactions, leading to increased antibody production. This, in turn, could drive immune complex formation that not only engages FcγR on myeloid cells, but also activates the complement cascade and is sensed by intracellular sensors such as TRIM21. These converging pathways may result in robust proinflammatory signalling, inflammasome activation, and cytotoxicity, collectively leading to the hyperinflammation and tissue damage characteristics of TB-IRIS. Previous candidate gene studies have implicated variants in *IL6* and *TNFA*, but these were not identified as top associations in our exome-wide screen, potentially reflecting differences in study design, population genetics, or the polygenic nature of the condition, where numerous variants of small effect contribute to risk. A more comprehensive GRS, developed and validated in larger, multi-ethnic cohorts, could help elucidate the genetic architecture of TB-IRIS and provide deeper insights into the biological pathways driving its pathogenesis.

## Figures and Tables

**Table 1:** Patient demographics and clinical characteristics. Eighty-two patients were randomly selected from the PredART trial for genetic analysis. Data are presented as median (Interquartile Range) for continuous variables and percentage (%) for categorical variables. P-values were generated comparing the two groups using the Mann-Whitney U test for continuous variables and Fisher’s exact test for categorical variables. Significant values are italicised and in bold. Abbreviations: ALT, alanine transaminase; AP, alkaline phosphatase; CRP, C-reactive protein; HIV VL, HIV viral load; INSHI, International Network for the Study of HIV-associated IRIS; IQR, interquartile range; WCC, white cell count.

	Non-IRIS (n=36)	TB-IRIS (n=46)	p-value
Age (median)	37 (IQR: 29-43)	35 (IQR: 30-41)	0.575
Sex (male)	22 (61%)	30 (65%)	0.701
Preventive Prednisone	9 (25%)	12 (26%)	0.911
TB-IRIS INSHI Fulfilment	N/A	41 (89%)	N/A
Time to TB-IRIS (day)	N/A	8 (IQR: 3-12)	N/A
Duration of TB-IRIS (day)	N/A	32 (IQR: 16-79)	N/A
Death	1	0	1.000
**Week 0**			
CD4 (cell/mL)	65 (IQR: 28-93)	31 (IQR: 22-62)	***0.016***
HIV VL (10^4^ copies/mL)	29.1 (IQR: 15.4-82.4)	29.3 (IQR: 16.9-59.3)	0.886
WCC (10^9^ cell/L)	3.7 (IQR: 2.7-5.3)	3.0 (IQR: 2.1-4.2)	0.065
AP (U/L)	106 (IQR: 88-138)	109 (IQR: 83-149)	0.847
ALT (U/L)	27.5 (IQR: 16.5-43.5)	33 (IQR: 21.8-44)	0.219
Haemoglobin (g/dL)	10.3 (IQR: 9.1-11.4)	10.1 (IQR: 8.5-10.8)	0.137
CRP (mg/L)	9.4 (IQR: 3.9-32.6)	8.3 (IQR: 4-19.2)	0.733
**Week 2**			
WCC (10^9^ cell/L)	5.3 (IQR: 4.5-6.6)	7.3 (IQR: 5.0-10.1)	***0.004***
AP (U/L)	107 (IQR: 87-149)	111 (IQR: 81-164)	0.822
ALT (U/L)	30 (IQR: 20.3-41.8)	27.5 (IQR: 22-53.5)	0.681
Haemoglobin (g/dL)	10.7 (IQR: 9.7-11.9)	10.4 (IQR: 9.4-11.6)	0.464
CRP (mg/L)	30.9 (IQR: 7.8-74.2)	67 (IQR: 52.7-130.2)	***0.0005***

## Data Availability

All sequencing data generated for this study have been deposited in NCBI databases. WES data are available in the SRA repository under accession number PRJNA1328844. RNA-Seq data are available in the GEO repository under accession number GSE274086
